# The Refraction of the Eye

**Published:** 1900-09

**Authors:** 


THE REFRACTION OF THE EYE. A Manual for Students. By GUSTAVUS HARTRIDGE, F. R. C. S., Senior Surgeon to the Royal Westminster Ophthalmic Hospital; Ophthalmic Surgeon and Lecturer on Ophthalmic Surgery to the Westminster Hospital; Ophthalmic Surgeon to St. Bartholomew's Hospital, Chatham; Consulting Ophthalmic Surgeon to St. George's Dispensary, Hanover Square, etc. With 105 illustrations. Tenth edition. Philadelphia: P. Blakiston's Son & Co., 1012 Walnut Street. 1900.

Hartridge's refraction of the eye has now reached its tenth edition, the first having being issued in 1884. As an elementary treatise on refraction it has deserved the favor it has received, both in England and America. It is clear and concise, and the author has kept abreast of the times. In this edition he has, as he says in the preface, carefully revised every page and made alterations in accordance with our increasing knowledge of the subject. The original plan of the work is preserved, which not only includes the consideration of errors of refraction, but strabismus and asthenopia are also discussed in their relations to refraction, and a chapter is added on spectacles. The author is conservative in his views, but he is not antiquated. It is a good work for a student in refraction to begin with. A. A. H.

